# Chemical Diversity in *Lippia alba* (Mill.) N. E. Brown Germplasm

**DOI:** 10.1155/2015/321924

**Published:** 2015-05-14

**Authors:** Arie Fitzgerald Blank, Lídia Cristina Alves Camêlo, Maria de Fátima Arrigoni-Blank, José Baldin Pinheiro, Thiago Matos Andrade, Edenilson dos Santos Niculau, Péricles Barreto Alves

**Affiliations:** ^1^Department of Agronomic Engineering, Federal University of Sergipe, Avenida Marechal Rondon s/n, 49100-000 São Cristóvão, SE, Brazil; ^2^Escola Superior de Agricultura “Luiz de Queiroz”, Universidade de São Paulo, Avenida Pádua Dias 11, Vila Independência, 13418-900 Piracicaba, SP, Brazil; ^3^Department of Chemistry, Federal University of Sergipe, Avenida Marechal Rondon s/n, 49100-000 São Cristóvão, SE, Brazil

## Abstract

The aim of this study was to perform chemical characterization of *Lippia alba* accessions from the Active Germplasm Bank of the Federal University of Sergipe. A randomized block experimental design with two replications was applied. The analysis of the chemical composition of the essential oils was conducted using a gas chromatograph coupled to a mass spectrometer. The chemical composition of the essential oils allowed the accessions to be allocated to the following six groups: group 1: linalool, 1,8-cineole, and caryophyllene oxide; group 2: linalool, geranial, neral, 1,8-cineol, and caryophyllene oxide; group 3: limonene, carvone, and sabinene; group 4: carvone, limonene, g-muurolene, and myrcene; group 5: neral, geranial, and caryophyllene oxide; and group 6: geranial, neral, o-cymene, limonene, and caryophyllene oxide.

## 1. Introduction

The Verbenaceae family consists of approximately 175 genera and 2,800 species distributed in tropical and subtropical regions worldwide and in temperate regions of the southern hemisphere. In addition, a few species are found in temperate regions of the northern hemisphere [1]. The genus* Lippia* includes several plant species of medicinal interest and comprises approximately 200 shrub species with a pantropical distribution and approximately 150 species distributed across rupestrian grasslands and tropical savannas (cerrados) in Brazil [2]. The species* Lippia alba* (Mill.) N.E. Brown, also known as* Lippia geminata* HBK and* Lantana alba* (Mill), is a shrub with a height of approximately 3 meters [3]. In traditional Brazilian medicine, this species is popularly known as lemon balm [4].

This species is considered by some authors [5] to be promising for use in the pharmaceutical, aromatic, and perfumery industries and may also be suitable for the agricultural chemical industry because of its proven antifungal, insecticidal, and repellent properties. The essential oil obtained from* L. alba* has been recognized as a potential source of several commercially important terpenoid compounds [6].

The chemical composition of essential oils consists of a mixture of many organic compounds in various concentrations, ranging from very low quantities (traces) to major compounds. Therefore, the variability of chemical types is a cause for concern from the standpoint of the use of essential oils as herbal medicines because some compounds may be unsuitable for achieving a desired result. This problem regarding medicinal plants is common in Brazil [7].

Essential oils are primarily produced by the plant leaves and are formed through the secondary metabolism of plants. The typical compounds of this type include mono- and sesquiterpenes. Both the oil composition and plant yield, including biomass, are directly influenced by environmental factors, which represent a challenge for producers in establishing productive and stable genotypes and maintaining the chemical uniformity demanded by the industry [5].

The rich pharmacological potential of* L. alba* is related to the wide chemical variability of its essential oils. This variability allows the classification of this species into chemotypes, which can be defined according to the major chemical components of the essential oils [8]. The essential oils of some chemotypes identified from* L. alba* differ in their chemical composition, with citral, carvone, and linalool representing the major components identified [9]. The limonene-carvone chemotype is characterized by the presence of limonene and carvone and the absence of neral and geranial (citral). Limonene is used as a solvent in cleaning products, foodstuffs, and the cosmetics industry. Carvone is used as a carminative and in cosmetic products and has bactericidal and fungicidal properties [10].

The degree of variability in the active ingredient found in a medicinal species should be very low so that the drugs produced from that species are safe and effective [8]. Therefore, the identification and correct classification of chemotypes, which exhibit distinct active ingredients in medicinal plants, are of great importance for maintaining quality, planning cultivation, and obtaining phytochemicals that do not impair users' health [11].

Thus, the aim of this study was to perform chemical characterization of accessions of* L. alba* (Mill.) NE Br. from the Active Germplasm Bank (AGB) of the Federal University of Sergipe.

## 2. Materials and Methods

### 2.1. Plant Materials

The experiment was conducted at the “Campus Rural da UFS” experimental farm, located in the municipality of São Cristóvão, state of Sergipe, at a latitude of 11°00′ S and longitude of 37°12′ W. Plants of 48 accessions of* L. alba* from the AGB at UFS were evaluated (Table 1).

A randomized block experimental design with two replications was employed. Each plot consisted of three plants. Spacing of 1.5 meters was maintained both between individual plants and between rows. The fertilization applied in the field was 5 kg of cattle manure per pit. Culture practices such as weeding and hydration were performed whenever necessary.

### 2.2. Distillation and Analysis of Essential Oils

The plants were cut at a height of 30 cm from the soil, and the leaves were dried in an incubator with forced airflow at a temperature of 40°C for five days [12]. After drying, the leaves were weighed on an electronic scale, and essential oils were extracted using the hydrodistillation method in a Clevenger apparatus. For hydrodistillation, 75 g of dry leaves and 2.0 L of distilled water were used per flask, and the distillation period was 120 minutes after the initiation of water vapor condensation in the Clevenger apparatus. After extraction, the essential oils were collected and stored in a freezer in amber glass vials.

Chemical analysis of the essential oil was performed at the Laboratory of Chromatography of the Department of Chemistry at the Federal University of Sergipe.

Qualitative analysis of the chemical composition of the essential oil was performed in a gas chromatograph coupled to a mass spectrometer (GC-MS, model QP 5050A, Shimadzu) equipped with an AOC-20i autosampler (Shimadzu) and a fused-silica capillary column (5% phenyl, 95% dimethylpolysiloxane, 30 m × 0.25 mm i.d., and film thickness of 0.25 *μ*m, J&W Scientific) using helium as the carrier gas at a flow rate of 1.2 mL min^−1^. The temperature ramp was 50°C for 2 min, followed by an increase of 4°C min^−1^ until reaching 200°C, then an increase of 15°C until reaching 300°C, after which a constant temperature was maintained for 15 min. The injector temperature was maintained at 250°C, and that of the detector (or interface) was maintained at 280°C. A volume of 0.5 *μ*L was injected using ethyl acetate. The partition rate of the injected volume was 1 : 100, and the column pressure was 64.20 kPa. The MS conditions included an ion capture detector operated through electron impact and an impact energy of 70 eV, a scan rate of 1,000, a scan interval of 0.50 fragment/s, and a fragment mass range between 40 Da and 500 Da.

Quantitative analysis of the chemical constituents was performed by flame ionization gas chromatography (FID), using a Shimadzu GC-17A (Shimadzu Corporation, Kyoto, Japan) instrument, under the following operational conditions: capillary ZB-5MS column (5% phenyl-arylene-95%-dimethylpolysiloxane) fused-silica capillary column (30 m × 0.25 mm i.d. × 0.25 *μ*m film thickness) from Phenomenex (Torrance, CA, USA), under the same conditions as reported for the GC-MS. Quantification of each constituent was estimated by area normalization (%). Compound concentrations were calculated from the GC peak areas and they were arranged in order of GC elution.

The essential oil components were identified by comparing their mass spectra with the available spectra in the equipment database (NIST05 and WILEY8). Additionally, the measured retention indices were compared with those in the literature [13]. The relative retention indices (RRI) were determined using the van den Dool and Kratz [14] equation and a homologous series of *n*-alkanes (C_8_–C_18_) injected under the chromatography conditions described above.

### 2.3. Statistical Analysis

The data were subjected to variance analysis, and the means were compared using the Scott-Knott test (*p* ≤ 0.05). The chemical composition data were analyzed through two multivariate analysis methods: principal component analysis (PCA) and arrangement analysis (cluster) based on the similarity and distribution of the compounds, using Statistica software, version 7.0.

## 3. Results and Discussion

Among the compounds present in the essential oils from 48 accessions, 33 were identified and are listed according to their order of elution (Table 2).

The variation in the concentrations of compounds among the accessions may have been a consequence of their origin, considering that in this experiment all of the accessions were grown in the same environment. Similar results were observed previously in studies with three chemotypes of* L. alba* from different Brazilian states (Rio de Janeiro, Ceará, and São Paulo) [15] and with accessions of* Pogostemon* sp. from the Active Germplasm Bank of the Federal University of Sergipe [16].

**Figure 1:**
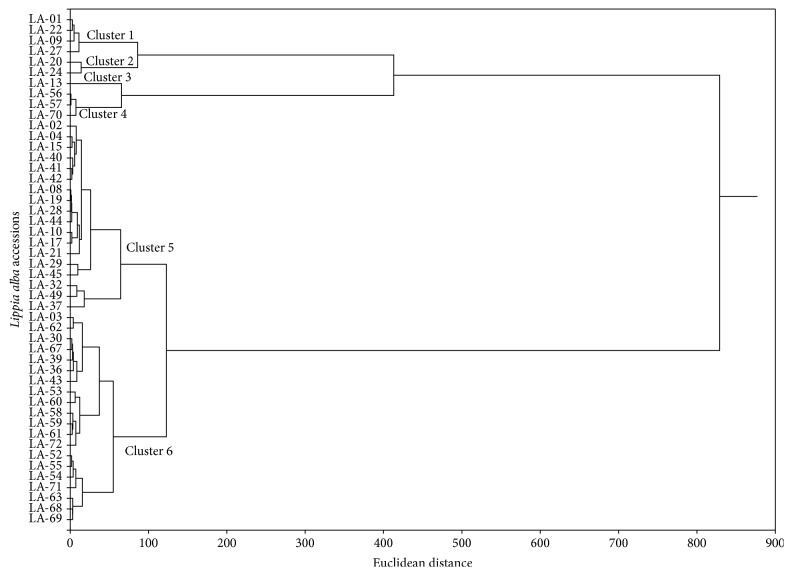
Two-dimensional dendrogram showing the similarity of the chemical compositions of 48* L. alba* accessions obtained from the Active Germplasm Bank at the Federal University of Sergipe.

The chemical analysis (Table 2) indicated that the most abundant compounds among the accessions were 1,8-cineole, linalool, myrcene, limonene, carvone, geranial, and neral, leading to the formation of six clusters according to the obtained chemical compositions, which were differentiated through cluster analysis (Figure 1). The compound linalool, present in accessions LA-01 and LA-22 (84.73% and 84.45%, resp.), showed the highest abundance. A similar result was reported by [17]. Linalool is widely used in the perfume, cosmetic, and fragrance industries [12].

**Figure 2:**
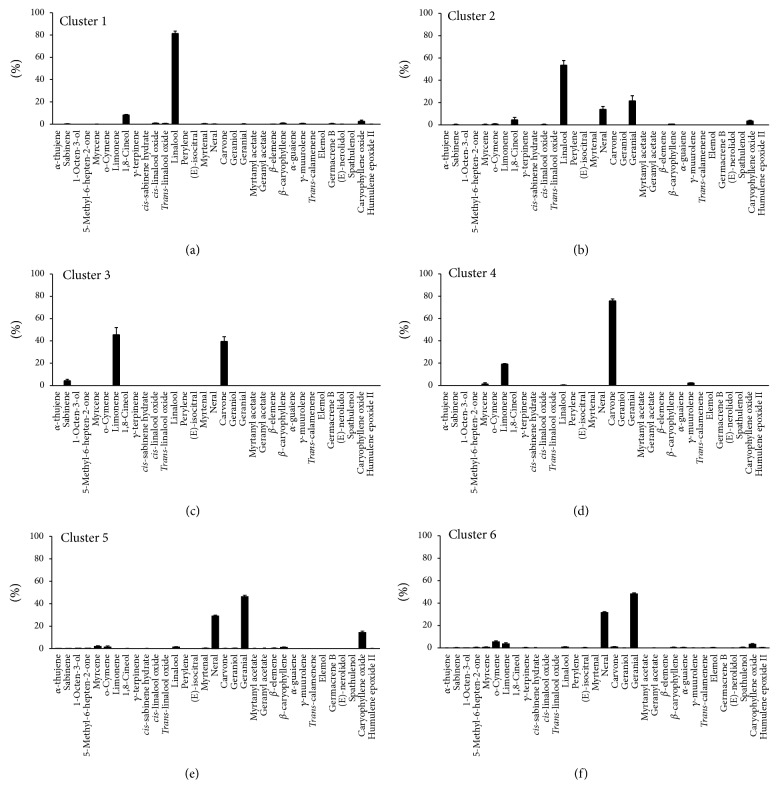
Means and SEM for the chemical compounds in the essential oils from clusters 1 through 6 of* L. alba* accessions.

Considering the similarities of the chemical constituents of the essential oils of these 48 accessions, the clusters were classified as follows: Cluster 1 included LA-01, LA-09, LA-22, and LA-27, which contained the following major compounds: linalool, 1,8-cineole, and caryophyllene oxide. Cluster 2 was formed by accessions LA-20 and LA-24 and comprised the following major compounds: linalool, geranial, neral, 1,8-cineole, and caryophyllene oxide. Cluster 3 was formed by accession LA-13, with the following major compounds: limonene, carvone, and sabinene. Cluster 4 comprised accessions LA-56, LA-57, and LA-70, with the following major compounds: carvone, limonene, g-muurolene, and myrcene. Cluster 5 was formed by accessions LA-02, LA-04, LA-08, LA-10, LA-15, LA-17, LA-19, LA-21, LA-28, LA-29, LA-32, LA-37, LA-40, LA-41, LA-42, LA-44, LA-45, and LA-49 and exhibited the following major compounds: neral, geranial, and caryophyllene oxide. Cluster 6 consisted of accessions LA-03, LA-30, LA-36, LA-39, LA-43, LA-52, LA-53, LA-54, LA-55, LA-58, LA-59, LA-60, LA-61, LA-62, LA-63, LA-67, LA-68, LA-69, LA-71, and LA-72, which contained geranial, neral, o-cymene, limonene, and caryophyllene oxide (Figure 2).

**Figure 3:**
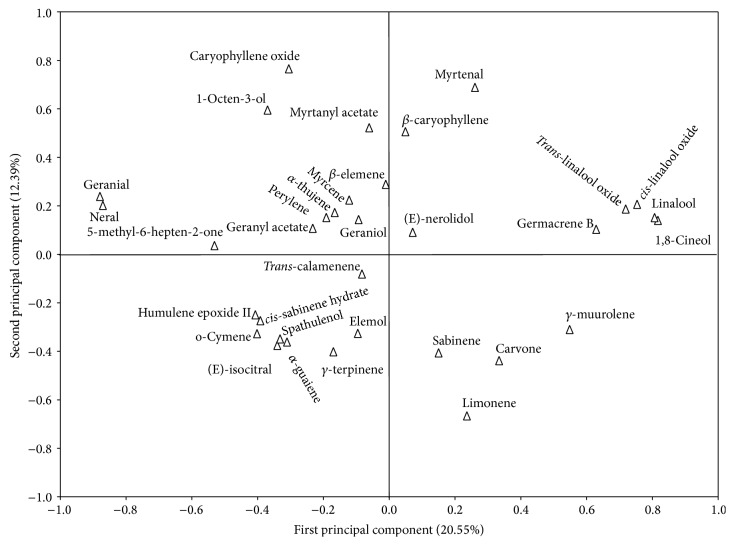
Distribution of chemical compounds in the essential oils of* L. alba* in relation to the first and second principal components, based on the principal component analysis (PCA).

According to the PCA (Figure 3), the first principal component accounted for 20.55% of the total variability in the data and was positively associated with 1,8-cineol (*r* = 0.82),* cis*-linalool oxide (*r* = 0.79), linalool (*r* = 0.82), and* trans*-linalool oxide (*r* = 0.77) and negatively associated with geranial (*r* = 0.90) and neral (*r* = −0.89).

The second principal component represented 12.39% of the total variation in the data and showed a positive correlation with 1-octen-3-ol (*r* = 0.60), myrtenal (*r* = 0.69), myrtanyl acetate (*r* = 0.54), and caryophyllene oxide (*r* = 0.75) and a negative correlation with carvone (*r* = −0.49) and limonene (*r* = −0.63).

## 4. Conclusions

Variability was observed in the chemical composition of the essential oils of* L. alba* accessions obtained from the AGB at UFS, resulting in the formation of six different groups.

The compounds characterizing the six groups were as follows: group 1: linalool, 1,8-cineole, and caryophyllene oxide; group 2: linalool, geranial, neral, 1,8-cineol, and caryophyllene oxide; group 3: limonene, carvone, and sabinene; group 4: carvone, limonene, g-muurolene, and myrcene; group 5: neral, geranial, and caryophyllene oxide; and group 6: geranial, neral, o-cymene, limonene, and caryophyllene oxide.

The most abundant compounds were 1,8-cineol, linalool, myrcene, limonene, carvone, geranial, and neral.

## Figures and Tables

**Table 1:** Identification and origin of *L. alba* accessions from the Active Germplasm Bank at the Federal University of Sergipe.

Accession	Municipality/state	Origin	UFS herbarium code
LA-01	ABC-Distrito Federal	University of Brasília	14784
LA-02	Araguaína-Tocantins	University of Brasília	14785
LA-03	Atibaia-São Paulo	University of Brasília	13466
LA-04	Botucatu-São Paulo	University of Brasília	13501
LA-08	Brasília-Distrito Federal	University of Brasília	13475
LA-09	Brasília-Distrito Federal	University of Brasília	14786
LA-10	Brasília-Distrito Federal	University of Brasília	13495
LA-13	Fortaleza-Ceará	Federal University of Ceará	13488
LA-15	Florianópolis-Santa Catarina	University of Brasília	13486
LA-17	Brasília-Distrito Federal	University of Brasília	13494
LA-19	Brasília-Distrito Federal	University of Brasília	13491
LA-20	Ilhéus-Bahia	University of Brasília	14787
LA-21	Brasília-Distrito Federal	University of Brasília	13493
LA-22	Lavras-Minas Gerais	University of Brasília	13476
LA-24	Luziânia-Goiás	University of Brasília	13477
LA-27	Piracicaba-São Paulo	University of Brasília	13443
LA-28	Brasília-Distrito Federal	University of Brasília	13487
LA-29	Planaltina de Goiás-Goiás	University of Brasília	13485
LA-30	Posse-Goiás	University of Brasília	13454
LA-32	Rio de Janeiro-Rio de Janeiro	University of Brasília	13480
LA-36	Brasília-Distrito Federal	University of Brasília	13472
LA-37	Brasília-Distrito Federal	University of Brasília	13455
LA-39	Brasília-Distrito Federal	University of Brasília	13497
LA-40	Brasília-Distrito Federal	University of Brasília	13456
LA-41	Curitiba-Paraná	University of Brasília	13484
LA-42	Brasília-Distrito Federal	University of Brasília	13444
LA-43	Brasília-Distrito Federal	University of Brasília	13490
LA-44	Brasília-Distrito Federal	University of Brasília	14788
LA-45	Viçosa-Minas Gerais	University of Brasília	13498
LA-49	Aracaju-Sergipe	Federal University of Sergipe	13471
LA-52	Rio Real-Bahia	Federal University of Sergipe	13481
LA-53	Telha-Sergipe	Federal University of Sergipe	13446
LA-54	Rio Real-Bahia	Federal University of Sergipe	13478
LA-55	Rio Real-Bahia	Federal University of Sergipe	13468
LA-56	Rio Real-Bahia	Federal University of Sergipe	13465
LA-57	Rio Real-Bahia	Federal University of Sergipe	13469
LA-58	Rio Real-Bahia	Federal University of Sergipe	13482
LA-59	Rio Real-Bahia	Federal University of Sergipe	13500
LA-60	Rio Real-Bahia	Federal University of Sergipe	13499
LA-61	Rio Real-Bahia	Federal University of Sergipe	13479
LA-62	Rio Real-Bahia	Federal University of Sergipe	13451
LA-63	Santana do São Francisco-Sergipe	Federal University of Sergipe	13445
LA-67	Santana do São Francisco-Sergipe	Federal University of Sergipe	13464
LA-68	Santana do São Francisco-Sergipe	Federal University of Sergipe	14789
LA-69	Gararu-Sergipe	Federal University of Sergipe	13467
LA-70	Cristinápolis-Sergipe	Federal University of Sergipe	13473
LA-71	Paripiranga-Sergipe	Federal University of Sergipe	13447
LA-72	Traipú-Alagoas	Federal University of Sergipe	13496

**Table 2:** Chemical composition of the essential oil of *L. alba* accessions from the Active Germplasm Bank at the Federal University of Sergipe.

Compound	RRI	Accession							

		LA-01	LA-02	LA-03	LA-04	LA-08	LA-09	LA-10	LA-13

*α*-thujene	924	0.00b	0.00b	0.43b	0.27b	0.00b	0.00b	0.00b	0.00b
Sabinene	969	0.00c	0.00c	0.00c	0.15c	0.00c	0.64b	0.00c	4.39a
1-Octen-3-ol	974	0.00b	0.00b	0.00b	0.28b	0.72a	0.00b	0.00b	0.00b
5-Methyl-6-hepten-2-one	981	0.00a	0.00a	0.46a	0.41a	0.34a	0.00a	0.00a	0.00a
Myrcene	988	0.00c	0.76c	2.78b	2.85b	0.31c	0.00c	0.00c	0.00c
o-Cymene	1022	0.00d	0.00d	0.11d	0.32d	0.00d	0.00d	0.00d	0.00d
Limonene	1024	0.00e	0.00e	10.24c	0.17e	0.00e	0.00e	0.00e	45.35a
1,8-Cineol	1026	7.22b	0.00d	0.00d	0.00d	0.00d	9.22a	0.00d	0.00d
*γ*-terpinene	1054	0.00c	0.00c	0.00c	0.00c	0.00c	0.00c	0.00c	0.00c
*cis*-sabinene hydrate	1065	0.00c	0.00c	0.00c	0.00c	0.00c	0.00c	0.00c	0.00c
*cis*-linalool oxide	1067	0.86b	0.00c	0.00c	0.00c	0.00c	1.20a	0.00c	0.00c
*trans*-linalool oxide	1084	0.65b	0.00d	0.00d	0.00d	0.00d	0.98a	0.00d	0.00d
Linalool	1095	84.73a	0.84f	0.89f	1.09f	0.95f	80.63b	1.32f	0.00f
Perylene	1102	0.00b	0.00b	0.10a	0.19a	0.00b	0.00b	0.00b	0.00b
(E)-isocitral	1177	0.00b	0.00b	0.50a	0.22b	0.00b	0.00b	0.00b	0.00b
Myrtenal	1195	0.67b	0.00c	0.29c	0.54b	0.56b	0.71b	0.00c	0.00c
Neral	1235	0.00f	32.14a	29.78c	30.28c	31.09c	0.00f	29.32c	0.00f
Carvone	1239	0.00e	0.00e	3.91d	0.33e	0.43e	0.00e	0.00e	39.58c
Geraniol	1249	0.00c	0.00c	0.71c	0.00c	0.00c	0.00c	0.00c	0.00c
Geranial	1264	0.00f	54.05a	47.66a	49.76a	49.45a	0.00f	48.06a	0.00f
Myrtanyl acetate	1324	0.00d	0.00d	0.13c	0.00d	0.24c	0.00d	0.00d	0.00d
Geranyl acetate	1379	0.00c	0.00c	0.35b	0.29b	0.00c	0.00c	0.82a	0.00c
*β*-elemene	1389	0.00d	0.00d	0.00d	0.00d	0.00d	0.00d	0.49d	0.00d
*β*-caryophyllene	1417	1.04c	0.42c	0.00c	0.78c	0.94c	1.11c	0.62c	0.00c
*α*-guaiene	1437	0.00d	0.00d	0.00d	0.00d	0.00d	0.00d	0.00d	0.00d
*γ*-muurolene	1478	0.62c	0.00c	0.29c	0.00c	0.00c	0.52c	0.00c	0.00c
*trans*-calamenene	1521	0.00b	0.00b	0.00b	0.00b	0.00b	0.00b	0.00b	0.00b
Elemol	1548	0.00d	0.00d	0.00d	0.00d	0.00d	0.00d	0.00d	0.00d
Germacrene B	1559	0.57b	0.00c	0.00c	0.00c	0.00c	0.28c	0.00c	0.00c
(E)-nerolidol	1561	0.00e	0.00e	0.00e	0.00e	0.00e	0.00e	0.00e	0.00e
Spathulenol	1577	0.00e	0.00e	0.00e	0.00e	0.00e	0.00e	0.00e	0.00e
Caryophyllene oxide	1582	2.93d	11.33c	0.00e	10.69c	14.24c	3.66d	17.77b	0.00e
Humulene epoxide II	1608	0.00d	0.00d	0.15d	0.00d	0.00d	0.00d	0.59b	0.00d
Essential oil content (%)		**2.53a**	**1.30d**	**2.06c**	**1.02d**	**0.79e**	**2.54a**	**0.86e**	**0.80e**

		LA-15	LA-17	LA-19	LA-20	LA-21	LA-22	LA-24	LA-27

*α*-thujene	924	0.00b	0.00b	0.00b	0.00b	0.00b	0.00b	0.00b	0.00b
Sabinene	969	0.00c	0.00c	0.00c	0.71b	0.00c	0.29c	0.00c	0.64b
1-Octen-3-ol	974	0.42a	0.78a	0.77a	0.00b	0.00b	0.00b	0.00b	0.00b
5-Methyl-6-hepten-2-one	981	1.03a	0.91a	0.58a	0.00a	0.38a	0.00a	0.00a	0.00a
Myrcene	988	3.07b	0.67c	0.45c	0.76c	0.84c	0.00c	0.00c	0.21c
o-Cymene	1022	0.00d	0.00d	0.00d	1.07d	0.00d	0.00d	0.62d	0.00d
Limonene	1024	0.00e	0.00e	0.00e	0.00e	0.00e	0.00e	0.00e	0.00e
1,8-Cineol	1026	0.00d	0.00d	0.00d	6.62b	0.93d	9.17a	2.38c	7.83b
*γ*-terpinene	1054	0.00c	0.00c	0.00c	0.00c	0.00c	0.00c	0.00c	0.00c
*cis*-sabinene hydrate	1065	0.00c	0.00c	0.00c	0.00c	0.00c	0.00c	0.00c	0.00c
*cis*-linalool oxide	1067	0.00c	0.00c	0.00c	0.64b	0.00c	1.00a	0.00c	0.80b
*trans*-linalool oxide	1084	0.00d	0.00d	0.00d	0.00d	0.00d	0.23c	0.00d	0.61b
Linalool	1095	0.83f	1.43f	0.83f	57.69d	7.27f	84.45a	49.38e	75.79c
Perylene	1102	0.00b	0.00b	0.00b	0.00b	0.00b	0.00b	0.00b	0.00b
(E)-isocitral	1177	0.00b	0.00b	0.00b	0.00b	0.00b	0.00b	0.00b	0.00b
Myrtenal	1195	0.00c	0.00c	0.95a	0.00c	0.34c	0.00c	0.00c	0.66b
Neral	1235	31.98a	28.91c	31.22c	11.51e	28.98c	0.00f	16.59e	0.58f
Carvone	1239	0.00e	0.00e	0.00e	0.00e	0.43e	0.00e	0.00e	0.00e
Geraniol	1249	0.00c	0.00c	0.00c	0.00c	0.00c	0.00c	0.00c	0.00c
Geranial	1264	50.54a	48.68a	49.09a	16.99e	44.91a	0.00f	26.18e	0.83f
Myrtanyl acetate	1324	0.00d	0.00d	0.53b	0.00d	0.00d	0.00d	0.00d	0.00d
Geranyl acetate	1379	0.00c	0.00c	0.00c	0.00c	0.00c	0.00c	0.00c	0.00c
*β*-elemene	1389	0.00d	0.25d	0.00d	0.00d	0.00d	0.00d	0.00d	0.46d
*β*-caryophyllene	1417	1.75b	1.03c	0.89c	0.83c	0.71c	0.29c	0.74c	1.59b
*α*-guaiene	1437	0.00d	0.00d	0.00d	0.00d	0.00d	0.00d	0.00d	0.00d
*γ*-muurolene	1478	0.00c	0.00c	0.00c	0.00c	0.00c	0.00c	0.00c	1.60b
*trans-*calamenene	1521	0.00b	0.00b	0.00b	0.00b	0.00b	0.00b	0.00b	0.00b
Elemol	1548	0.00d	0.00d	0.00d	0.00d	0.00d	0.00d	0.00d	0.00d
Germacrene B	1559	0.00c	0.00c	0.00c	0.00c	0.00c	0.00c	0.00c	1.26a
(E)-nerolidol	1561	0.00e	0.00e	0.00e	0.00e	0.00e	0.00e	0.00e	0.00e
Spathulenol	1577	0.00e	0.00e	0.00e	0.00e	0.00e	0.00e	0.00e	0.00e
Caryophyllene oxide	1582	10.35c	16.86b	14.04c	3.18d	14.63c	4.35d	4.11d	0.00e
Humulene epoxide II	1608	0.00d	0.00d	0.00d	0.00d	0.29c	0.20c	0.00d	0.00d
Essential oil content (%)		**1.09d**	**1.04d**	**0.89e**	**1.33d**	**0.76e**	**2.53a**	**2.50a**	**2.26b**

		LA-28	LA-29	LA-30	LA-32	LA-36	LA-37	LA-39	LA-40

*α*-thujene	924	0.00b	0.00b	0.00b	0.91a	0.00b	0.00b	0.00b	0.22b
Sabinene	969	0.00c	0.00c	0.27c	0.00c	0.24c	0.00c	0.53b	0.27c
1-Octen-3-ol	974	0.63a	0.00b	0.00b	0.31b	0.00b	1.19a	0.00b	0.63a
5-Methyl-6-hepten-2-one	981	0.00a	0.00a	0.72a	0.23a	0.46a	0.00a	0.35a	0.52a
Myrcene	988	0.00c	7.09a	0.40c	3.67b	0.17c	0.00c	0.20c	1.16c
o-Cymene	1022	0.00d	0.00d	3.71d	12.36a	3.96c	0.00d	5.30c	0.00d
Limonene	1024	0.00e	0.00e	7.08c	0.00e	6.80c	0.00e	8.55c	0.00e
1,8-Cineol	1026	0.00d	0.00d	0.00d	0.00d	0.00d	0.00d	0.00d	0.00d
*γ*-terpinene	1054	0.00c	0.00c	1.05a	0.00c	1.03a	0.00c	1.01a	0.00c
*cis*-sabinene hydrate	1065	0.00c	0.00c	0.00c	0.00c	0.00c	0.00c	0.00c	0.29b
*cis*-linalool oxide	1067	0.00c	0.00c	0.00c	0.00c	0.00c	0.00c	0.00c	0.30c
*trans*-linalool oxide	1084	0.00d	0.00d	0.00d	0.00d	0.00d	0.00d	0.00d	0.00d
Linalool	1095	0.91f	0.00f	0.92f	0.25f	0.85f	0.00f	0.92f	2.90f
Perylene	1102	0.00b	0.00b	0.00b	0.00b	0.00b	0.00b	0.00b	0.00b
(E)-isocitral	1177	0.00b	0.00b	0.00b	0.00b	0.34a	0.00b	0.57a	0.00b
Myrtenal	1195	0.72b	0.67b	0.00c	0.49b	0.00c	1.19a	0.00c	0.00c
Neral	1235	31.99a	28.09c	32.93a	21.94c	30.87c	23.76c	31.66a	29.95c
Carvone	1239	0.00e	0.56e	0.93e	0.39e	0.99e	1.16e	1.05e	0.00e
Geraniol	1249	0.00c	0.00c	0.00c	2.03b	0.00c	0.00c	0.00c	0.00c
Geranial	1264	49.73a	40.24c	49.07a	32.90c	51.32a	39.89c	48.00a	46.80a
Myrtanyl acetate	1324	0.00d	0.00d	0.00d	0.00d	0.00d	0.72a	0.00d	0.00d
Geranyl acetate	1379	0.00c	0.00c	0.00c	0.00c	0.00c	0.00c	0.00c	0.00c
*β*-elemene	1389	0.00d	1.19b	0.00d	0.00d	0.00d	0.00d	0.00d	1.88a
*β*-caryophyllene	1417	0.55c	0.00c	0.00c	3.69a	0.00c	1.54b	0.00c	1.31b
*α*-guaiene	1437	0.00d	0.00d	0.00d	0.00d	0.00d	0.00d	0.00d	0.00d
*γ*-muurolene	1478	0.00c	0.00c	0.41c	0.00c	0.40c	0.00c	0.00c	0.00c
*trans*-calamenene	1521	0.00b	0.00b	0.00b	0.00b	0.00b	0.00b	0.00b	0.00b
Elemol	1548	0.00d	0.00d	1.14c	0.00d	2.16a	0.00d	1.83a	0.00d
Germacrene B	1559	0.00c	0.00c	0.00c	0.00c	0.00c	0.00c	0.00c	0.00c
(E)-nerolidol	1561	0.00e	0.00e	1.14d	0.00e	2.16a	0.00e	1.83b	0.00e
Spathulenol	1577	0.00e	0.00e	0.00e	0.00e	0.00e	0.00e	0.00e	0.00e
Caryophyllene oxide	1582	14.86c	10.89c	1.32e	20.46b	0.00e	28.09a	0.00e	11.43c
Humulene epoxide II	1608	0.00d	0.00d	0.00d	0.35c	0.00d	0.00d	0.00d	0.00d
Essential oil content (%)		**0.58e**	**0.66e**	**1.06d**	**1.26d**	**2.21b**	**0.80e**	**1.84c**	**0.53e**

		LA-41	LA-42	LA-43	LA-44	LA-45	LA-49	LA-52	LA-53

*α*-thujene	924	0.00b	0.00b	0.00b	0.00b	0.00b	0.00b	0.00b	0.00b
Sabinene	969	0.14c	0.28c	0.31c	0.00c	0.98b	0.57b	0.54b	0.17c
1-Octen-3-ol	974	0.63a	0.44a	0.00b	0.58a	0.00b	0.21b	0.00b	0.59a
5-Methyl-6-hepten-2-one	981	0.74a	0.00a	0.43a	0.51a	0.30a	0.26a	0.30a	0.34a
Myrcene	988	1.47c	2.04c	0.71c	0.42c	8.47a	0.00c	0.21c	0.00c
o-Cymene	1022	0.31d	0.00d	0.00d	0.00d	0.32d	11.50a	9.44b	4.49c
Limonene	1024	0.00e	0.00e	7.46c	0.00e	0.00e	0.00e	3.86d	0.00e
1,8-Cineol	1026	0.00d	0.00d	0.00d	0.00d	0.00d	0.00d	0.00d	0.00d
*γ*-terpinene	1054	0.00c	0.00c	0.00c	0.00c	0.00c	0.00c	0.98a	0.00c
*cis*-sabinene hydrate	1065	0.00c	0.00c	0.00c	0.00c	0.46b	0.00c	0.00c	0.33b
*cis*-linalool oxide	1067	0.15c	0.00c	0.00c	0.00c	0.00c	0.27c	0.00c	0.00c
*trans*-linalool oxide	1084	0.00d	0.00d	0.00d	0.00d	0.00d	0.00d	0.00d	0.00d
Linalool	1095	0.85f	2.22f	0.74f	0.73f	1.33f	0.33f	0.00f	0.57f
Perylene	1102	0.00b	0.00b	0.00b	0.00b	0.00b	0.00b	0.00b	0.00b
(E)-isocitral	1177	0.00b	0.00b	0.75a	0.00b	0.38a	0.00b	0.46a	0.00b
Myrtenal	1195	0.00c	0.00c	0.00c	0.68b	0.00c	0.00c	0.00c	0.00c
Neral	1235	30.88c	31.33c	34.86a	32.27a	28.57c	23.69c	29.97c	31.57a
Carvone	1239	0.00e	0.00e	3.56d	0.00e	0.00e	0.00e	0.00e	0.00e
Geraniol	1249	0.00c	0.00c	0.00c	0.00c	3.38a	0.00c	0.00c	0.00c
Geranial	1264	48.69a	48.59a	51.18a	49.88a	46.36a	39.39c	46.22a	48.37a
Myrtanyl acetate	1324	0.00d	0.00d	0.00d	0.50b	0.00d	0.00d	0.00d	0.00d
Geranyl acetate	1379	0.27b	0.00c	0.00c	0.00c	0.40b	1.03a	0.00c	0.00c
*β*-elemene	1389	0.79c	1.75a	0.00d	0.00d	0.00d	0.00d	0.00d	0.00d
*β*-caryophyllene	1417	0.88c	1.23c	0.00c	0.00c	2.84a	1.15c	1.60b	0.00c
*α*-guaiene	1437	0.00d	0.00d	0.00d	0.00d	0.00d	0.00d	0.00d	1.66b
*γ*-muurolene	1478	0.00c	0.00c	0.00c	0.00c	0.33c	0.38c	0.00c	0.00c
*trans*-calamenene	1521	0.00b	0.00b	0.00b	0.00b	0.00b	0.00b	0.00b	0.00b
Elemol	1548	0.00d	0.00d	0.00d	0.00d	0.00d	0.00d	0.00d	0.00d
Germacrene B	1559	0.00c	0.00c	0.00c	0.00c	0.00c	0.00c	0.00c	0.00c
(E)-nerolidol	1561	0.00e	0.00e	0.00e	0.00e	0.00e	0.00e	0.00e	0.00e
Spathulenol	1577	0.00e	0.00e	0.00e	0.00e	0.00e	0.00e	0.00e	3.21a
Caryophyllene oxide	1582	12.46c	11.66c	0.00e	13.43c	5.37d	19.89b	6.05d	5.05d
Humulene epoxide II	1608	0.00d	0.00d	0.00d	0.00d	0.00d	0.66b	0.34c	1.45a
Essential oil content (%)		**0.85e**	**0.90e**	**1.80c**	**0.69e**	**1.06d**	**1.18d**	**2.00c**	**0.76e**

		LA-54	LA-55	LA-56	LA-57	LA-58	LA-59	LA-60	LA-61

*α*-thujene	924	0.00b	0.00b	0.00b	0.00b	0.00b	0.00b	0.00b	0.00b
Sabinene	969	0.59b	0.53b	0.00c	0.00c	0.00c	0.25c	0.00c	0.00c
1-Octen-3-ol	974	0.00b	0.00b	0.00b	0.00b	0.67a	0.52a	0.00b	0.00b
5-Methyl-6-hepten-2-one	981	0.00a	0.00a	0.00a	0.00a	0.65a	0.92a	0.00a	0.53a
Myrcene	988	0.00c	0.00c	0.17c	0.15c	1.27c	2.80b	0.00c	1.31c
o-Cymene	1022	9.55b	8.29b	0.00d	0.00d	2.93c	3.77c	6.47b	3.53c
Limonene	1024	3.97d	3.69d	18.48b	19.35b	0.00e	0.00e	2.60e	0.00e
1,8-Cineol	1026	0.00d	0.00d	0.00d	0.00d	0.00d	0.00d	0.00d	0.00d
*γ*-terpinene	1054	0.90a	0.93a	0.00c	0.00c	0.00c	0.00c	0.85a	0.00c
*cis*-sabinene hydrate	1065	0.00c	0.00c	0.00c	0.00c	0.00c	0.22c	0.00c	0.00c
*cis*-linalool oxide	1067	0.00c	0.00c	0.00c	0.00c	0.00c	0.00c	0.00c	0.00c
*trans*-linalool oxide	1084	0.00d	0.00d	0.00d	0.00d	0.00d	0.00d	0.00d	0.00d
Linalool	1095	0.00f	0.00f	0.32f	0.19f	2.83f	2.31f	0.00f	2.30f
Perylene	1102	0.00b	0.00b	0.00b	0.00b	0.14a	0.13a	0.00b	0.00b
(E)-isocitral	1177	0.85a	0.44a	0.00b	0.00b	0.00b	0.00b	0.00b	0.00b
Myrtenal	1195	0.00c	0.00c	0.00c	0.00c	0.00c	0.00c	0.00c	0.00c
Neral	1235	29.99c	30.98c	0.00f	0.00f	33.20a	33.93a	33.46a	35.31a
Carvone	1239	0.00e	0.00e	77.74a	77.20a	0.00e	0.00e	0.00e	0.00e
Geraniol	1249	0.00c	0.00c	0.00c	0.00c	0.00c	0.00c	0.00c	0.00c
Geranial	1264	44.02a	47.27a	0.00f	0.00f	51.42a	51.35a	48.61a	52.86a
Myrtanyl acetate	1324	0.00d	0.00d	0.00d	0.00d	0.00d	0.00d	0.00d	0.00d
Geranyl acetate	1379	0.00c	0.00c	0.00c	0.00c	1.01a	0.83a	0.00c	0.83a
*β*-elemene	1389	0.00d	0.00d	0.00d	0.00d	0.00d	0.00d	0.00d	0.00d
*β*-caryophyllene	1417	2.18b	1.88b	0.00c	0.00c	0.00c	0.00c	3.16a	0.00c
*α*-guaiene	1437	0.00d	0.00d	0.00d	0.00d	0.00d	0.00d	0.00d	0.00d
*γ*-muurolene	1478	0.00c	0.00c	1.99b	1.95b	0.00c	0.00c	0.00c	0.00c
*trans*-calamenene	1521	0.00b	0.00b	0.00b	0.00b	0.00b	0.00b	0.00b	0.00b
Elemol	1548	0.00d	0.00d	0.00d	0.00d	0.00d	0.00d	0.00d	0.00d
Germacrene B	1559	0.00c	0.00c	0.00c	0.00c	0.00c	0.00c	0.00c	0.00c
(E)-nerolidol	1561	0.00e	0.00e	0.00e	0.00e	0.00e	0.00e	0.00e	0.00e
Spathulenol	1577	0.00e	0.00e	0.00e	0.00e	0.00e	0.00e	0.00e	0.00e
Caryophyllene oxide	1582	7.54d	5.96d	0.00e	0.00e	5.00d	2.59d	4.85d	3.00d
Humulene epoxide II	1608	0.38c	0.00d	0.00d	0.00d	0.87b	0.36c	0.00d	0.31c
Essential oil content (%)		**1.93c**	**2.33b**	**1.60c**	**2.66a**	**0.66e**	**1.00d**	**1.73c**	**0.66e**

		LA-62	LA-63	LA-67	LA-68	LA-69	LA-70	LA-71	LA-72

*α*-thujene	924	0.00b	0.25b	0.00b	0.00b	0.00b	0.00b	0.24b	0.00b
Sabinene	969	0.00c	0.75b	0.00c	0.81b	0.37b	0.00c	0.55b	0.51b
1-Octen-3-ol	974	0.00b	0.59a	0.00b	0.00b	0.34b	0.00b	0.00b	0.17b
5-Methyl-6-hepten-2-one	981	0.46a	0.85a	0.00a	0.44a	0.81a	0.00a	0.28a	1.01a
Myrcene	988	5.07b	0.00c	0.00c	0.00c	0.00c	3.40b	0.20c	0.00c
o-Cymene	1022	0.00d	8.97b	4.61c	10.33a	11.86a	0.00d	11.39a	0.81d
Limonene	1024	9.14c	0.00e	7.98c	0.28e	0.00e	19.81b	4.21d	0.00e
1,8-Cineol	1026	0.00d	0.00d	0.00d	0.00d	0.00d	0.00d	0.00d	0.00d
*γ*-terpinene	1054	0.00c	0.00c	0.86a	0.00c	0.00c	0.00c	0.47a	0.00c
*cis*-sabinene hydrate	1065	0.00c	0.61a	0.00c	0.73a	0.35b	0.00c	0.00c	0.74a
*cis*-linalool oxide	1067	0.00c	0.00c	0.00c	0.00c	0.00c	0.00c	0.00c	0.00c
*trans*-linalool oxide	1084	0.00d	0.00d	0.00d	0.00d	0.00d	0.00d	0.00d	0.00d
Linalool	1095	0.58f	0.99f	0.43f	1.06f	0.63f	0.69f	0.27f	0.48f
Perylene	1102	0.00b	0.00b	0.00b	0.00b	0.00b	0.00b	0.00b	0.00b
(E)-isocitral	1177	0.63a	0.98a	0.44a	0.00b	0.24b	0.00b	0.77a	0.58a
Myrtenal	1195	0.00c	0.00c	0.00c	0.00c	0.00c	0.00c	0.00c	0.00c
Neral	1235	31.36c	29.46c	33.28a	27.34c	29.05c	0.00f	27.94c	36.14a
Carvone	1239	5.27d	0.00e	1.06e	0.00e	0.29e	72.73b	0.00e	0.00e
Geraniol	1249	0.00c	0.00c	0.00c	0.00c	0.00c	0.00c	0.00c	0.00c
Geranial	1264	46.11a	45.12a	49.53a	45.36a	45.21a	0.00f	42.06c	54.63a
Myrtanyl acetate	1324	0.00d	0.00d	0.00d	0.00d	0.00d	0.00d	0.00d	0.00d
Geranyl acetate	1379	0.00c	0.00c	0.00c	0.00c	0.00c	0.00c	0.00c	0.64a
*β*-elemene	1389	0.00d	0.00d	0.00d	0.00d	0.00d	0.00d	0.00d	0.00d
*β*-caryophyllene	1417	0.39c	0.00c	0.00c	0.00c	0.00c	0.00c	1.20c	0.00c
*α*-guaiene	1437	0.00d	1.36c	0.00d	2.89a	1.58b	0.00d	0.00d	0.00d
*γ*-muurolene	1478	0.00c	0.00c	0.26c	0.00c	0.00c	2.57a	0.00c	0.00c
*trans*-calamenene	1521	0.00b	0.00b	0.00b	0.00b	0.00b	0.00b	0.00b	1.24a
Elemol	1548	0.00d	0.00d	1.54b	0.00d	0.00d	0.00d	0.00d	0.00d
Germacrene B	1559	0.00c	0.00c	0.00c	0.00c	0.00c	0.00c	0.00c	0.00c
(E)-nerolidol	1561	0.00e	0.00e	1.54c	0.00e	0.00e	0.00e	0.00e	0.00e
Spathulenol	1577	0.00e	2.85b	0.00e	2.59c	1.82d	0.00e	0.00e	0.00e
Caryophyllene oxide	1582	0.99e	4.10d	0.00e	3.58d	4.59d	0.00e	9.28c	1.57e
Humulene epoxide II	1608	0.00d	1.17a	0.00d	1.33a	0.36c	0.00d	0.71b	0.00d
Essential oil content (%)		**1.59c**	**0.66e**	**2.80a**	**1.23d**	**1.17d**	**1.95c**	**1.46d**	**1.20d**

RRI: relative retention index. Means followed by different letters in each row were significantly different by the Scott-Knott test (*p* < 0.05).
